# Challenges in and opportunities for individualising diabetes technology: a position statement by the European Association for the Study of Diabetes (EASD) and the American Diabetes Association (ADA) Diabetes Technology Working Group

**DOI:** 10.1007/s00125-025-06651-9

**Published:** 2026-03-12

**Authors:** Daniela Bruttomesso, John R. Petrie, Mark Evans, G. Alexander Fleming, Helene Hanaire, Reinhard W. Holl, Jennifer L. Sherr, Richard M. Bergenstal, Lutz Heinemann, Anne L. Peters

**Affiliations:** 1 Unit of Metabolic Diseases, Department of Medicine, University of Padova, Padova, Italy; 2 School of Health and Wellbeing, College of Medical, Veterinary and Life Sciences, University of Glasgow, Glasgow, UK; 3 Institute of Metabolic Science, University of Cambridge, Cambridge, UK; 4 Kinexum, Harpers Ferry, WV USA; 5 Department of Diabetology, University Hospital of Toulouse, University of Toulouse, Toulouse, France; 6 Institute of Epidemiology and Medical Biometry, ZIBMT, University of Ulm, Ulm, Germany; 7 German Center for Diabetes Research (DZD), Munich-Neuherberg, Germany; 8 Yale University School of Medicine, New Haven, CT USA; 9 International Diabetes Center and HealthPartners Institute, Minneapolis, MN USA; 10 Science & Co, Düsseldorf, Germany; 11 Keck School of Medicine and the Leonard D. Schaeffer Institute for Public Policy & Government Service of the University of Southern California, Los Angeles, CA USA

**Keywords:** Access, Automated insulin delivery, Barriers, Challenges, Diabetes technology, Digital divide, Disparities, Opportunities, Type 1 diabetes, Type 2 diabetes

## Abstract

From fingerstick blood glucose monitoring and mechanical insulin pens in the 1970s to modern automated insulin delivery systems, rapidly progressing advances in diabetes technology are transforming management options for people with diabetes, particularly those with type 1 diabetes but also, increasingly, people with type 2 diabetes. However, access to life-changing diabetes technologies is neither uniform nor universally covered, and there is no one size fits all approach. In this position statement, we emphasise to healthcare professionals the importance of supporting individuals with diabetes to access and use the right diabetes technology according to personal needs, capabilities and preferences. In doing so, we highlight the equal importance of avoiding disparities in the provision of diabetes technology by challenging preconceived barriers, which can be overcome with education and determination. We also make a series of suggestions for action to advance the more widespread adoption of diabetes technology while minimising the ‘digital divide’.

## Introduction

Diabetes technology has become a pillar of diabetes management and is recommended for use in many settings for people with diabetes [1–3], but individual needs must be accounted for to provide and optimise a personalised treatment approach. Individualisation of care requires knowledge of the wants, needs and preferences of each person with diabetes. At the same time, it is critical to recognise the impact of social determinants of health [4] and the ‘digital/device’ divide that exists between adequately served and underserved individuals, even in countries in which access to care is publicly funded and free at the point of use [5–7].

This presents a challenge for clinicians, who have to stay informed and up to date on the characteristics, advantages and limitations of each device, drawing from both clinical trials and real-world experience, and match these features to each individual’s changing needs and conditions (e.g. lipohypertrophy, skin reactions, pregnancy, cognitive function, digital literacy, diabetes distress, burnout, psychosocial stressors and access to care).

Clinicians need to consider whether a proposed technology satisfies therapeutic goals developed jointly with the person living with diabetes, while taking into account availability, affordability, ease of use and accessibility. In this position statement, we outline the current landscape of individualised diabetes therapies, highlighting both opportunities and challenges. In addition, we present suggestions for action, along with definitions of outcomes and barriers that must be addressed.

The Diabetes Technology Working Group was established by the EASD and ADA in 2013. The group reached agreement on the suggestions for actions through a small number of face-to-face meetings (usually at large diabetes conferences), many online meetings and numerous emails. This is the last in a series of comparable articles by the group over the last 10 years.

## Supporting people with diabetes to select diabetes technology

Medical devices should be tailored to individual needs and preferences [1]. To optimise the likelihood of user satisfaction and therapeutic success, clinical teams need to work with people with diabetes to explain and discuss the attributes (Tables 1 and 2) and efficacy of different devices [8–10]. Any discrepancies in expectations should be worked through in frank conversations, setting realistic expectations for what can be achieved with technology. Identifying the right device for an individual is likely to increase adherence, which in turn may translate into better glycaemic management and/or improved quality of life. In this way, the costs associated with device acquisition and use are more likely to be offset by the benefits associated with the prevention of acute and long-term diabetes complications and/or the decreased burden of living with the condition.

**Table 1:**
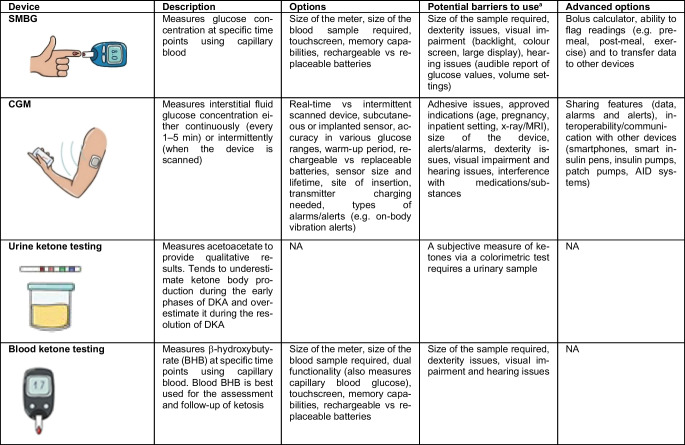
Features to consider when selecting optimal diagnostic diabetes technologies

^a^For each device, consider if it is covered by insurance, affordable, accessible or approved

DKA, diabetic ketoacidosis; NA, not available

**Table 2:**
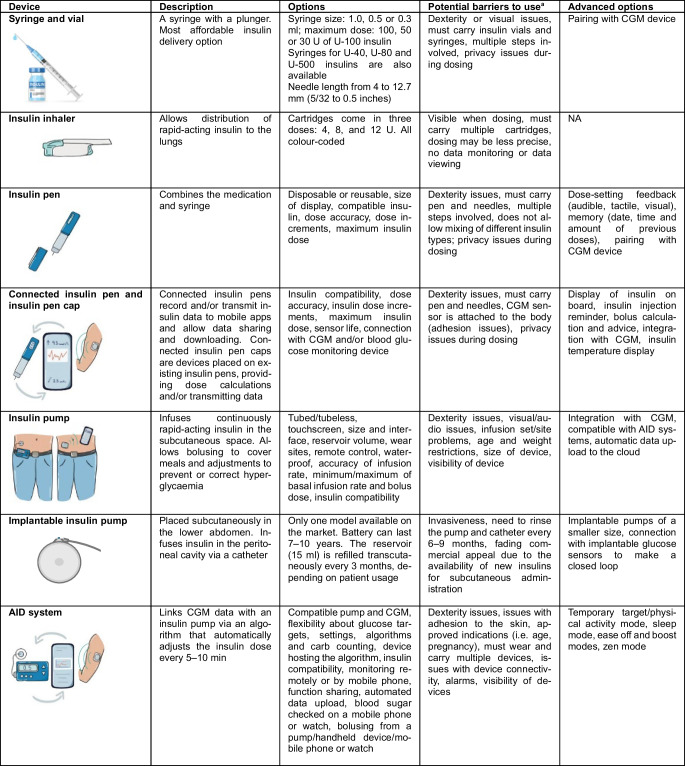
Features to consider when selecting optimal insulin delivery devices

^a^For each device, consider if it is covered by insurance, affordable, accessible or approved

NA, not available

The choice of a (new) device normally requires face-to-face or telemedicine contact. Interactions with healthcare professionals can be augmented with reputable online educational resources provided by diabetes charities, patient organisations, scientific bodies and device manufacturers. However, health and digital literacy, as well as proficiency in the language in which the materials are provided, will affect the ability of people with diabetes to benefit from these resources. Where possible, information on diabetes technology should be available in the primary language of the individual concerned [11]. Increasingly, materials can also be provided in the form of infographics and video content that has been co-developed by people with diabetes. Adjuncts to written educational resources can include social media discussions in which experts and users of specific diabetes technologies share their personal experiences with devices. Such discussions can add considerable value but need to be curated. Bias is common, as people who are most happy or unhappy with a device are more likely to post their opinions. Furthermore, manufacturer incentives may not be transparent. Specialised healthcare professionals should maintain some awareness of the content of social media platforms so that they can discuss them with people with diabetes and correct any inaccuracies.

Factors to consider in choosing the most appropriate diabetes technology for an individual with diabetes include the type of diabetes, age, personal preferences (e.g. for a tubeless device), quality of current glucose management, current total daily insulin dose, personalised glucose target, lifestyle, level of education, manual dexterity, digital literacy, occupation (including any specific risks), comorbidities, socioeconomic status, psychosocial factors, access to medical care, and device(s) already used (Table 3) [12]. It is also important to consider an individual’s physical and cognitive status, as well as whether they are ready for a change. For people whose diabetes is partially or wholly managed by someone else (i.e. young children or people with cognitive impairments), the caregivers’ skills and preferences also need to be considered [1].


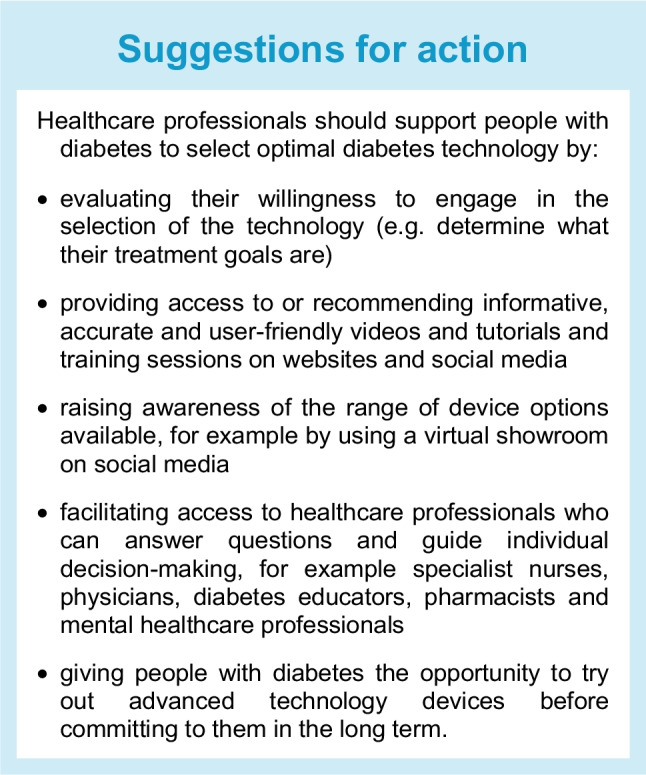


**Table 3:** Individualisation of diabetes technology: considerations and solutions

Factor	Consideration	Solution
Type of diabetes	Certain devices are covered through insurance based on the type of diabetes and/or whether or not the person with diabetes is on insulin	Understand whether or not a diabetes technology is covered for an individual; OTC CGM is available for people not on insulin in some places who can afford it and who do not need alarms
Current level of control	People with diabetes with very high glucose levels may need levels to be lowered into the target range slowly to avoid symptoms and possible worsening of retinopathy	Gradually introduce diabetes technology as indicated. The use of systems that have variable targets can be helpful
Readiness for change	Introducing a new form of technology requires cooperation between the person with diabetes and the provider	Agreeing on the stepwise addition of technology can be helpful. Insisting on change is not beneficial, but working with an individual over time and understanding their concerns and beliefs can be helpful
Lifestyle/occupation	People with diabetes often have lifestyle considerations that impact device choice, e.g. participation in contact sports, prolonged and frequent time spent in the water, and occupational constraints	Review the activities carried out by the individual with diabetes to help guide decision making. Work to devise approaches to aid in success, such as over-bandages and device placement. Where sanitation is not readily available at a worksite, help the individual think through ways to clean hands (e.g. portable wipes) and infusion sites if necessary
Age	Diabetes technology is often approved only for certain age groups	Ensure that the individual with diabetes fits the criteria for a device. Be aware of the ages indicated in regulatory approvals. When appropriate, consider pursuing prior authorisation for off-label use
Level of education/digital literacy	Assess the familiarity of the individual with diabetes with technology generally and their preferred learning approach/ability to learn through written materials	Provide learning tools appropriate for an individual’s level of need. Offer various modalities for learning, such as videos, individual sessions, group classes and peer support
Physical limitations	Any injury/condition that impacts the use of both hands/arms, including arthritis, stenosing tenosynovitis, amputation, injury or congenital anomalies	Work with the individual and/or caregiver to determine which diabetes technologies can be used, either independently or with assistance. In some cases an individual can use diabetes technology but not insert it themselves; this can be performed by a caregiver
Access to technology/internet	Rural and underserved urban communities can lack access compared with their urban counterparts. Not all individuals, even in the same city, have the same access	Work through solutions such as using a receiver/reader, going to a local site such as a library to upload data and participating in local efforts to provide better access in areas where it is lacking (available in some locations)
Comorbidities	For example, reduced vision, dialysis, hearing loss, gastroparesis and reduced cognitive function	Assess each individual with diabetes on a case-by-case basis, choosing a system they can use most effectively and creating settings that provide the most benefit with the least disruption. Consider the caregiver role as indicated
Socioeconomic status/access to medical care	Barriers related to socioeconomic status/access to medical care should be assessed and efforts made to provide consistent care regardless	Identify what each individual with diabetes can afford and assess barriers such as co-pays and complicated authorisation processes. Ensure that each person with diabetes has the basics for survival, even if they must default to a lower-tech solution
Language	Diabetes technology does not come ready to use in all languages, and the guides/videos/tools for training are not available in all languages	Attempt to find a device trainer fluent in the language spoken by the individual with diabetes; if on-device instructions are not available in the necessary language, provide whatever training/tools the individual needs to use the diabetes technology safely
Caregiver management	If caregivers are involved in patient care, they should have a role in choosing the appropriate diabetes technology	Caregivers should be trained and have clear protocols for diabetes technology use. This is particularly important when caregivers change and when the care of an individual is provided by multiple caregivers. All caregivers should be trained in the use of each type of diabetes technology employed and understand how to troubleshoot and who to call if difficulties arise
Psychosocial concerns/diabetes distress	People with diabetes often have diabetes distress, anxiety, depression and other psychosocial concerns, which can impact their use of diabetes technology	Screen for mental health issues, such as depression and diabetes distress, at least annually and more often as needed. Ensure access to a behavioural health individual/team for assessment and treatment. Provide people with diabetes with a choice of diabetes technology and support their decisions (such as taking a ‘pump break’) so they are always managed safely

## Types of diabetes and diabetes technology

### Type 1 diabetes and diabetes technology

Diabetes technology has transformed the management of type 1 diabetes over the last 50 years, starting with the availability of personal blood glucose meters in the 1970s, followed by insulin pumps (continuous subcutaneous insulin infusion or CSII) since the 1990s [13], continuous glucose monitoring (CGM) systems since the 2000s [14] and automated insulin delivery (AID) systems since 2016 [15], which use algorithms to automatically adjust basal insulin delivery based on CGM measurements. These diabetes technologies have allowed people with diabetes who have access to appropriate devices to make data-driven decisions to improve glucose management and reduce the daily burden of diabetes.

However, challenges remain, as advanced technologies have high acquisition costs (even if cost-effective) and are not accessible to all with type 1 diabetes, even in higher income countries [16]. This highlights the need for more affordable options and far-sighted decisions by healthcare systems (irrespective of their own specific funding models). Reorganisation of healthcare delivery may be required to ensure that people with diabetes receive proper education on new technologies and integrate them correctly into daily life (e.g. re-designation of healthcare professional roles and use of remote and/or group education). For example, in Scotland, additional administrative staff have been secured from the National Health Service (NHS) so that healthcare professionals can use their time more efficiently in the roll out of the next stage of technology, and all users of conventional CSII have the chance to upgrade to AID systems [17]. This is a good example of helping healthcare professionals on board individuals with diabetes to diabetes technology.

A growing body of evidence indicates that diabetes technology improves glycaemic outcomes in type 1 diabetes, increasing the time in range (TIR) and the likelihood of meeting other clinical targets across all age groups and socioeconomic backgrounds [18, 19]. Of the available technologies, AID systems are best for the regulation of blood glucose levels, with dual-hormone full closed-loop systems being particularly effective in preventing hypoglycaemia in individuals who are within the target range for glycaemic management and have a longer diabetes duration [19]. The use of AID systems also reduces diabetes distress compared with usual care, without increasing the risk of adverse events, both in adults and in children [20].

Current AID systems rely on carbohydrate counting to achieve optimal glucose management; however, adequate glucose management can also be reached using semi-quantitative carbohydrate estimation [21].

### Type 2 diabetes and diabetes technology

Until recently, individuals with type 2 diabetes had fewer diabetes technology options, but access to CGM technology is currently increasing, particularly for those who are insulin treated. A key driver for widening access to diabetes technology among people with type 2 diabetes is the growing evidence of benefit in this large patient group. Indications for CGM have been expanding, from people with type 2 diabetes who require multiple daily insulin injections with frequent blood glucose monitoring [22] to those requiring less complex regimens, including basal insulin [23], and even those not on insulin therapy [1, 24–27]. Additionally, AID systems appear to be effective in people with type 2 diabetes on insulin therapy.

In some countries, such as the USA, modified versions of CGM are available for over-the-counter (OTC) use. These devices do not have the alarms or alerts of the prescription-requiring devices and may be preset for different glucose ranges; however, they are effective for people who wish to pay out of pocket and monitor their glucose levels. Increasing data exist to guide individuals and clinicians as to whether CGM data reflect normal or abnormal glucose tolerance [28, 29]. Additionally, studies are beginning to show benefits from the use of CGM in lifestyle programmes, often in conjunction with other wearable technologies [30–32].

Recent meta-analyses have shown that, compared with self-monitoring of blood glucose (SMBG), the use of CGM is associated with better glucose management in people with type 2 diabetes, both insulin and non-insulin treated [33–36]. When consistent use of CGM is not feasible, periodic use of personal or professional CGM may be considered to adjust medication and/or lifestyle [1]. A combination of CGM and SMBG may also be used. For example, a CGM system can be used for 2 weeks out of every 3 months, preferably just before a visit with a healthcare provider [26]. When not using CGM, SMBG can be used as needed. This can provide feedback on glucose changes in response to food and exercise, as well as provide summary numerical assessments of glycaemic management.

Recently, promising results have been obtained using AID in the treatment of type 2 diabetes. The improvements observed in study participants, in terms of both glucose levels and psychological traits, mirrored the results seen in people with type 1 diabetes: lower HbA_1c_ levels, increased TIR without increasing the risk of hypoglycaemia, reduction in distress related to diabetes and improved quality of sleep. The superiority of AID was independent of participant age and comparator regimen (multiple daily injections [MDI], basal insulin or oral glucose-lowering medications, including glucagon-like peptide-1 receptor agonists [GLP-1RAs] and sodium–glucose cotransporter 2 [SGLT2] inhibitors) and was seen across diverse racial, ethnic and socioeconomic backgrounds [37–43]. In people with type 2 diabetes, AID may also mitigate clinical inertia and ease the burden of insulin dose intensification when glycaemic targets are not met (whether with insulin alone or with insulin plus non-insulin glucose-lowering agents) [43].


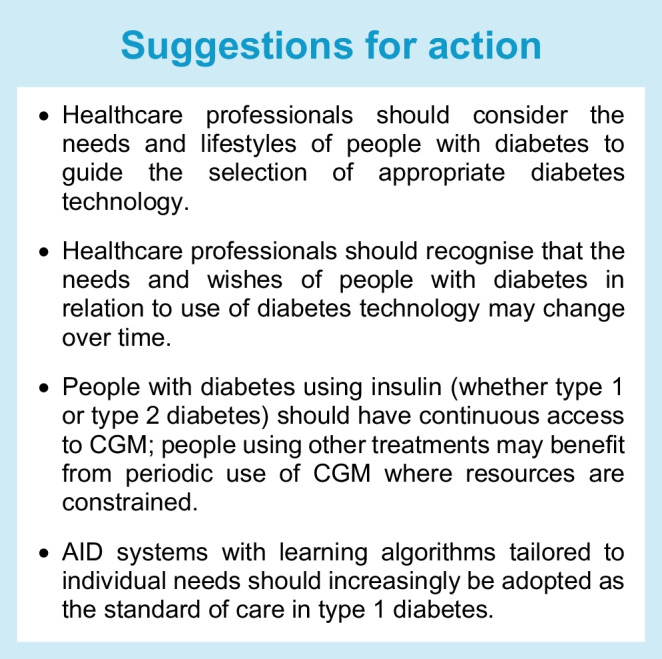


### Special populations

#### People at high risk of hypoglycaemia

Diabetes technology can benefit individuals with long-standing type 1 diabetes and impaired awareness of hypoglycaemia (IAH), a condition that affects 20–40% of people with type 1 diabetes and increases the risk of severe hypoglycaemia. Both CGM and AID reduce the frequency and magnitude of hypoglycaemic events, although an elevated risk of severe hypoglycaemia in IAH persists [44], underscoring an ongoing need for educational initiatives and improved treatment strategies. At present, it is unclear whether CGM and AID restore awareness of hypoglycaemia or simply decrease the frequency of hypoglycaemic episodes via alarms [45].

#### Older people

Older adults with type 1 diabetes, who are frequently affected by comorbidities, are at greater risk of severe hypoglycaemia and its consequences, including changes in cognition, seizures, falls and fractures [46]. Use of diabetes technology by older people is growing, with a remarkable impact on diabetes-related outcomes and quality of life. In particular, CGM has been shown to increase TIR, reduce time below range (TBR) and decrease the risks of hypoglycaemia and hospitalisation [46, 47]. In some studies, CGM has also been shown to have a positive impact on psychological outcomes, patient satisfaction and quality of life [48]. Concomitant use of smart insulin pens and CGM has the potential to improve adherence to insulin dosing, detect accidental incorrect dosing and reduce the risk of severe hypoglycaemia [49]. Smart pens also represent an excellent option for residents of long-term care facilities who manage their diabetes under supervision [46].

In this population, insulin pump therapy may also mitigate glucose variability and reduce the risk of hypoglycaemia, but more data are needed on the use of insulin pumps by older people with comorbidities, cognitive problems and an inability to cope with emergencies (including pump malfunction) [46, 48]. Several trials have addressed the use of AID in older people with diabetes; for example, compared with sensor augmented pumps, hybrid closed-loop systems, have been shown to decrease HbA_1c_, increase TIR, decrease TBR and hypoglycaemia, reduce the diabetes-related psychological burden and increase self-confidence in managing diabetes during exercise [50–52]. Future studies should focus on how the new technologies can be used by older adults with cognitive defects or who are non-autonomous, especially in the context of long-term care facilities [53].

#### Pancreatectomy

Because of a lack of both insulin and glucagon, diabetes after total pancreatectomy presents with higher glucose variability and an increased risk of hypoglycaemia. A recent meta-analysis indicated that CGM used in the perioperative period alone or in combination with a pump improved glycaemic management and decreased the risk of hypoglycaemia in this population [54]. In a single case study, after problems (including severe hypoglycaemia) with several other modalities of insulin delivery, a pancreatectomised individual was switched to AID, reaching an acceptable level of glucose management [55]. In a short, randomised crossover clinical trial, a bi-hormonal AID system (i.e. administering both insulin and glucagon) provided superior glycaemic outcomes to insulin pump and pen therapy without increasing the rate of adverse events and without requiring inputs related to meals or physical activity [56]. Larger randomised trials that include formal assessment of quality of life and diabetes distress as outcomes are needed to assess the long-term effectiveness and safety of AID systems after pancreatectomy.

#### Cystic fibrosis

In addition to endocrine and exocrine pancreatic insufficiency, people with diabetes and cystic fibrosis have increased rates of intermittent infections and experience frequent use of steroids and chronic inflammation [57]. A recent meta-analysis and systematic review of non-randomised studies reported that the weighted mean difference in HbA_1c_ was 4.1 mmol/mol (0.4%) lower with CGM than with SMBG, albeit with a high risk of bias [58]. Studies on the use of insulin pumps in cystic fibrosis are few and based on old models. Recently, a small, randomised crossover trial found that, with respect to usual care, AID use in cystic fibrosis improved glucose management, with more episodes of self-reported symptomatic hypoglycaemia [59].

#### Islet cell/pancreas transplantation

Beta cell replacement (pancreatic islet/pancreas transplantation) is indicated for people with type 1 diabetes with significant glycaemic variability and a higher risk of severe hypoglycaemia despite optimised management, or who are unable to successfully engage with diabetes technology [2, 60]. These procedures can provide a period of insulin independence of varying, sometimes limited, duration. Following beta cell replacement failure, the use of AID may restore glucose management, but further studies are needed to establish whether AID may represent an alternative to re-transplantation [61].

## Economic benefits and financial considerations

Like all health interventions, diabetes technologies have a cost encompassing financial, physical and emotional aspects. Each diabetes technology prescription (or selection by a person with diabetes) requires a choice, ideally made jointly between the person with diabetes and their healthcare team. Acquisition and running costs are likely to influence decision making in different ways depending on whether they are paid directly by a state-funded healthcare system, covered by healthcare insurance and/or paid for out of pocket by the person with diabetes. ‘Co-pay’ systems may limit access for some individuals. The complexity of a device and the training required for its optimal usage also exert an influence.

Increasing evidence has shown that the use of diabetes technology is associated with significant cost savings and reduced rates of emergency department visits and hospitalisations. For example, economic benefits have been seen with use of AID systems in people with type 1 diabetes [62, 63]. Reductions in hospitalisation rates and emergency department visits have also been seen with use of CGM in people with type 2 diabetes [64, 65]. Reduced hospitalisation rates for acute diabetes events and cardiovascular complications have been observed in adults with type 1 diabetes using CGM in Sweden, and there is similar evidence from other healthcare systems, including the Canadian private payer system [66], Medicaid in the USA [64] and the French health system [67], and from Swedish and Australasian registries [68–71].

Despite the evidence-based benefits of diabetes technology, coverage for devices is variable depending on the country and even the district of residence. For example, recent policy changes in Australia have permitted examination of how coverage of devices can impact care at a population level. Government funding for CGM was approved in April 2017 for people with type 1 diabetes under 21 years of age. Before this change, ~5% of this population were using CGM, but this increased to 79% 2 years after the subsidy became effective. Concomitant with the adoption of CGM was an increase in the odds of achieving an HbA_1c_ <53 mmol/mol (7%), which persisted during the 2 year follow-up period [70]. Subsequently, a cost analysis highlighted that government-subsidised CGM was cost-effective compared with user-funded access to CGM [71].

The evidence base that exists today supporting the cost-effectiveness of diabetes technologies is summarised in the standards of care from the ADA, consensus reports from the ADA and EASD on the management of type 1 diabetes in adults, and clinical practice guidelines from the International Society for Pediatric and Adolescent Diabetes. These guidelines provide language that can be used to champion the expansion of access to diabetes technology for people with diabetes who can benefit [1, 2, 72]. For example, they can be used to support healthcare professionals in discussions with insurers on individual cases. Indeed, through the efforts of multiple stakeholders in the UK, for example, guidance from the National Institute for Health and Care Excellence recommends the use of hybrid closed-loop AID systems to support children and adults with type 1 diabetes to achieve glycaemic targets (or avoid hypoglycaemia) provided these are procured at a cost-effective price by the NHS from the manufacturing companies [62].

## Data analysis/interpretation

There have been considerable advances towards harmonising glucose data display among CGM devices. The ambulatory glucose profile (AGP) provides a template format [73, 74] and is now becoming the industry standard. However, in our clinical experience there may be large differences in how both people with diabetes and healthcare professionals interpret data. At present, there remain nuances of presentation (and even colour coding) that may limit full interoperability. For example, some people with type 1 diabetes worry about discrepancies between fingerstick readings and CGM readings and revert to the old methods. Others find CGM useful in avoiding hypoglycaemia, in avoiding foods that result in ‘spikes’ or in monitoring mean glucose levels, but still struggle to use the data to make appropriate adjustments to their basal or bolus insulin doses. The situation is not dissimilar for healthcare professionals, who are on a spectrum of knowledge/expertise and numeracy. Some with training or experience in data science can quickly grasp the visual information presented in the AGP, but others may be more reliant on summary statistics and derived parameters. A stepwise approach to CGM interpretation is advised, that is, the person reviewing the data should check the quality of the data (e.g. percentage of time that the CGM was active) before making suggestions on treatment adjustment. In their standards of care, the ADA states that ‘Some authors also suggest approaches to changing therapy plans based on the data reviewed that enable healthcare professionals to make a simple yet comprehensive review and plan of care even within the time constraints of office visits’ [1]. For this reason, some manufacturers provide automated decision aids with suggestions for insulin adjustment (albeit with disclaimers in the user agreements covering potential harms). For the full benefits of CGM to be derived for all, more research is required to evaluate how people with type 1 diabetes and healthcare professionals interpret and react to CGM data, how interpretations are influenced by the method of data presentation and the reproducibility of such interpretations within and between individuals. Such research should assess whether there are systematic differences between specific groups of healthcare professionals (e.g. doctors, nurses, dietitians) and people with diabetes (e.g. children, adolescents, older people) in different contexts (e.g. pregnancy, community, inpatient, clinic) and how such insights can be used to tailor training and education for individuals.

## Defining optimal outcomes

As traditionally defined, the optimal outcomes in diabetes care involve multiple targets: glucose levels, blood pressure, lipid levels, cardiovascular risk and diabetes-related complications. Many factors must be considered for an individual trying to reach such evidence-based goals and targets, including the extent of diabetes distress and burden, access to care, social determinants of health and personal preferences. Therefore, to achieve optimal outcomes, the focus should shift towards personalised care (e.g. ‘How can the available diabetes technology best be matched to each individual with diabetes?’ and ‘Which factors determine whether an individual can use diabetes technology, and which devices should be considered?’) rather than crude metrics (‘X% of my patients are on pumps’ or ‘Y% of our patients are on sensors’). In addition, where resources are limited, it is clearly appropriate to ask who will benefit most from the use of diabetes technology. Such a shift in focus speaks to reaching shared diabetes technology goals co-developed by people with diabetes and their clinicians.

### Access and disparities

As mentioned above, acquisition costs and prices of consumables for diabetes technology can be high, and there are costs associated with initiating and training people with diabetes on using diabetes technology and interpreting data. Unfortunately, in many places, this has created a digital divide in knowledge and access to technology as well as in the availability of diabetes educators and training. This can inadvertently worsen healthcare disparities and lead to a lower rate of use of diabetes technology in the people who need it most (e.g. in ethnic minority and other underserved populations) [75–79].

To ensure that access to devices is equitable, it is critical for healthcare providers to be aware of potential biases, sometimes subconscious, that may exist and impact prescribing patterns (Table 3) [76, 77, 79].

Even in a national healthcare system such as the NHS in the UK, which is free at the point of delivery with no out-of-pocket costs for people with diabetes, individuals who are better educated or privileged and/or more knowledgeable about the healthcare system generally advocate more effectively for themselves so that, as coverage is extended, they tend to gain access to newer diabetes technologies more readily than those of lower educational and socioeconomic status [7, 80–82].

Programmes have been developed to help increase diabetes technology use by marginalised populations, with some success. However, models of care require staff to be flexible and spend time obtaining appropriate devices for individuals and educating them on their use [83, 84]. Successful projects may include involving healthcare professionals beyond the immediate diabetes team (e.g. community pharmacists) or changing the roles of existing staff [85]. For example, it has been shown that educating older people on diabetes technology use takes longer than educating younger individuals but is achievable if sufficient time is spent [86]. Where flexibility and/or reallocation of roles cannot be offered, these present significant barriers. There are published examples of initiatives that have been successful in reducing the digital divide [87, 88].

Inequality of access to diabetes technology, and indeed any sort of specialised diabetes care, is a critical topic. A study in the UK demonstrated that retinopathy was less common in those on CSII than in those who were eligible for CSII but continued on MDI; however, CSII users were noted to be from less socially deprived areas [89]. Such observations reflect complex societal issues. In addition, disparities remain for diverse demographic groups and people with physical impairments, including impaired manual dexterity or visual impairment [90]. Other factors that can impact access to diabetes technology include non-supported languages, health/digital literacy issues and differences in access to mobile phone signals and/or the internet. Finally, refugees/immigrants lack access to diabetes technology regardless of where they are located in the world.

Historically, people with diabetes needed to be deemed ‘appropriate candidates’ to be prescribed diabetes technology. Such evaluations are, of course, subjective, whether derived from perceptions of the frequency of visits with diabetes care providers, engagement with glucose monitoring, ability to benefit from group structured education or demonstration of basic knowledge about diabetes management. With the ability to examine subpopulations in larger trials and real-world datasets, it is clear that baseline HbA_1c_ and socioeconomic status should not be used to identify those most appropriate for device use [91, 92]. ‘One-to-one starts’ should be made available for those who cannot easily work in group settings or who require additional educational and technical support. Finally, there is anecdotal evidence that people with diabetes may prefer devices that are closer to their own skin colour. Manufacturers should therefore consider offering a variety of options to improve user satisfaction.

Just as the WHO has identified essential medications, including insulin and glucagon, for people with diabetes, we envision the evidence-based creation of an essential technologies list, which should be publicly available and accessible anywhere. While the ultimate goal is to provide access to the most advanced and current diabetes technologies, it is critical to understand that there are settings, particularly in low- and middle-income countries, where individuals can only afford/be provided with test strips and a glucose meter. In these settings, it is not feasible to test blood glucose levels as often as would be optimal. A degree of pragmatism is required on the part of healthcare professionals, championing equity of access but approaching each individual in their own right (e.g. HbA_1c_ targets may need to be higher for some people with diabetes so that they can be as healthy and safe as possible within the healthcare environment in which they reside).


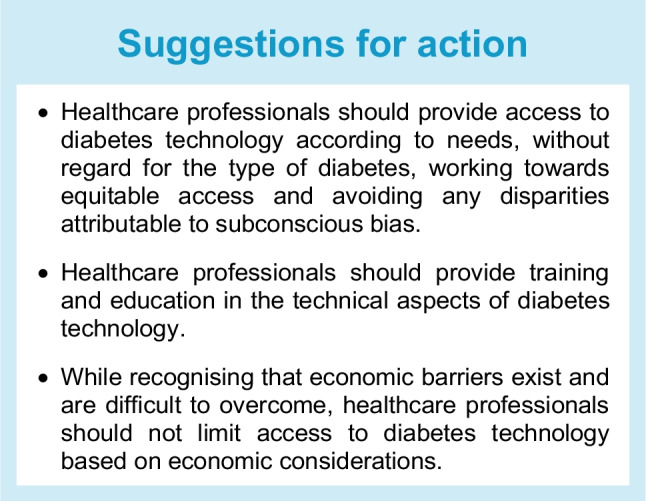


### Physical concerns: skin issues

To ensure proper functioning, glucose sensors must remain securely attached to the skin, especially during periods of high activity (i.e. exercise, manual work, contact sports). If a sensor detaches, it may be necessary to wait for a new sensor, as the number of sensors permitted in a given time period may be capped by an individual’s insurance coverage or healthcare system. In this regard, healthcare professionals should try to find ways of permitting flexibility and avoiding out-of-pocket expenses.

Skin irritation (e.g. erythema and itching) from the adhesive used to fix a device (or from the plastic housing of the device itself) has been reported by up to one-third of individuals with diabetes and may lead to discontinuation [93–95]. Devices used for diabetes treatment can also cause skin injury (e.g. allergic/hypersensitivity reactions, contact dermatitis) or scarring with long-term use. Research and innovation are needed in this domain (e.g. use of alternative ‘hypoallergenic’ adhesives). Implanted CGM devices can overcome some of the skin issues, although a transmitter does need to be placed on top of the sensor and occasionally the device fails and needs to be removed and re-inserted [96].

Devices that infuse insulin can cause lipohypertrophy [97], and continuous use of the same location can make insulin absorption erratic. Research is ongoing to better characterise device–dermis interactions and how to optimise insulin delivery [98].

Approaches to prevent and treat dermatological reactions to diabetes technology vary but often require trial and error. Suggestions based on clinical experience have been published and can also be found on various websites [99, 100].

### Psychosocial considerations

#### People with diabetes: psychological barriers

Although diabetes technology helps people with diabetes achieve their glucose targets and often has a positive impact on psychosocial outcomes, its adoption can be limited by psychological barriers including diabetes distress, diabetes burnout, unrealistic expectations and the impacts of depression, anxiety and attention deficit hyperactivity disorder (ADHD) on self-care [101, 102]. Continuous device use can make diabetes more visible to others, potentially leading to feelings of embarrassment or self-consciousness [103]. Some people with diabetes may experience a sense of being ‘tethered’ to their devices or may express concerns about appearing overly medicalised or ‘robotic’ in social settings [104]. Anecdotally, tubeless pumps may be more acceptable to such individuals; their availability should be highlighted (particularly to adolescent patients) and such devices should be included in the range of diabetes technology offered at the time of initiation.

Parents of children with diabetes have expressed fears of physical discomfort and interference with physical activity in their children, which they perceive may outweigh the advantages of diabetes technology [105]. Concerns around physical intimacy have also been expressed by adolescents and adults [106]. Additionally, worry related to the insertion of the cannula for an insulin pump infusion set can temper enthusiasm for adoption of this device, which is also the case for the replacement of glucose sensors in CGM systems. Finally, the quantity of information provided by glucose monitors can be overwhelming, which may impact initiation and persistence of device use [103, 105]. Such concerns may best be addressed by discussing the wealth of evidence supporting the beneficial impact of diabetes technology.

Newer studies assessing AID systems have often shown improvements in psychological outcomes and quality of life [107–109] and self-reported state, anxiety and guilt [110], while not completely abolishing diabetes distress [38, 111]. Several surveys [112–114] have also shown the persistence of psychosocial symptoms in some individuals using diabetes technology. This may be because there is always a burden to device use; even when using a device, managing diabetes still requires time, input and attention.

In some cases, there may be a need to include a clinical psychologist when deciding on the use of advanced technologies in diabetes [115]. However, it is always up to the individual with diabetes to decide what is best, given that they will be wearing the device 24/7. People can trial a device or switch from one system to another, depending on coverage. It may be necessary to take a temporary break from technology, which is acceptable as long as there is a safe back-up plan in place for diabetes management.

#### Healthcare professionals: psychological barriers

Healthcare professionals can find it difficult to stay up to date with the rapid developments in diabetes technology; they require education and time for continuing professional development [103]. Within a large diabetes team, it may be appropriate for some healthcare professionals to focus on people with diabetes using advanced diabetes technology, while others remain responsible for educating team members on developments and building confidence.

As discussed above, implicit biases that induce inequity in access to diabetes technology must be actively combated. Some healthcare professionals may perceive that people with diabetes dislike wearing devices, that diabetes technology may be too complicated for them or their families or that technology will increase the time spent on diabetes management [116]. There may also be implicit biases that restrain healthcare professionals from proposing diabetes technology to people with diabetes who they may consider not to be ‘good’ candidates, perhaps because of their age, low education level or low income. It is critical to understand the consequences of such biases, whether implicit or explicit [117].

The perception of an individual’s device readiness should be openly discussed with the individual to limit the risk of denying access to technology [104, 118].


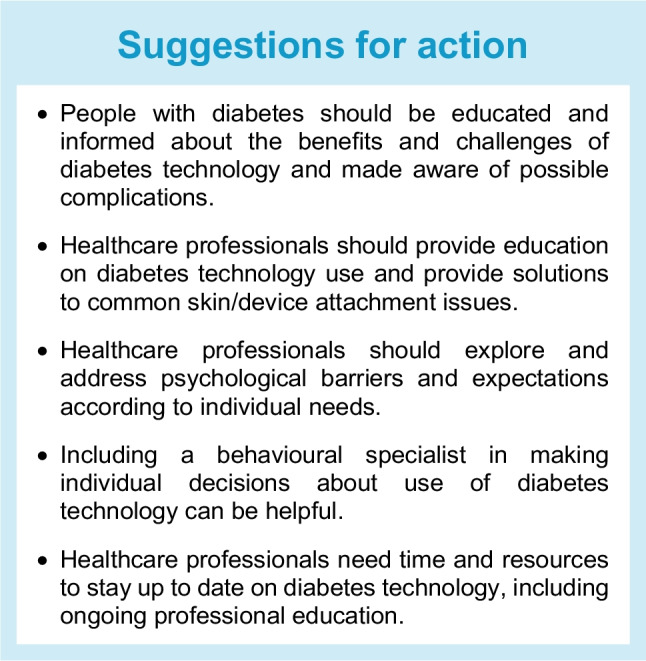


## Safety

Even with modern technology systems, errors can occur, for example insulin pump failure, insulin precipitation and infusion set obstruction, problems at the infusion site, user error or a combination of these issues. Pump failure can be due to mechanical problems or to software malfunction and entails an increased risk of ketoacidosis and hypoglycaemia [119, 120]. CGM devices can be inaccurate, lose connectivity and/or stop working. Moreover, AID systems that use algorithms that are one size fits all may be less safe for individuals who habitually undertake extreme or intermittent exercise.

Thus, safety should be considered when matching people with diabetes to diabetes technology and there must be adequate training and follow-up, as well as training for healthcare professionals. Even though many of the insulin dosing decisions are made automatically, providing a person with diabetes with the best hardware is only one step in the process of achieving an optimal clinical outcome. Training and education for people with diabetes should be provided by healthcare professionals according to the level of technology offered [21, 121, 122]. Additional support will be required for individuals who are unable to read; efforts should be made to personalise patient education and understanding of materials (typically, healthcare information is provided in a format suitable for people who can read at a higher level).

The automation behind AID systems lowers the level of understanding required to manage diabetes and optimise treatment; however, this only holds true if the technology always works perfectly. As this is not the case in daily life, people with diabetes need to have access to back-up strategies and be able to solve problems when specialist support is less available. For example, if there is inadequate understanding of a concept such as ketone testing, a reaction to a technical issue might not be sufficient and might result in further harm.


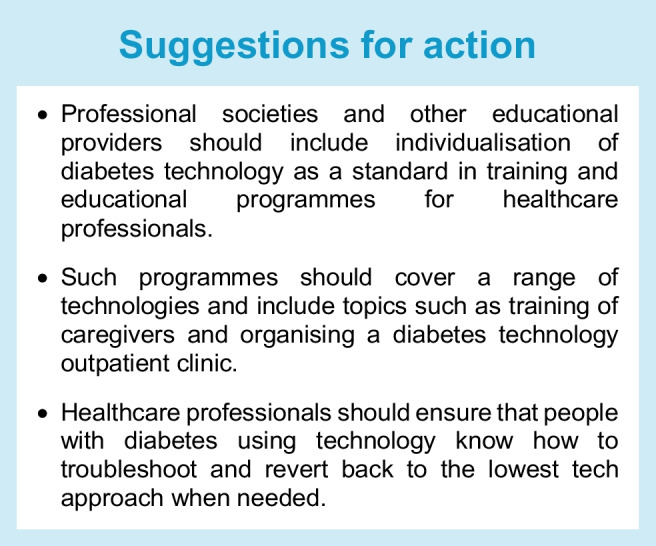


## Role of remote monitoring technology for virtual diabetes care

The potential of remote monitoring in diabetes care was discussed long before the COVID-19 pandemic, particularly for those who need more frequent follow-up or who live far from major centres. However, previously, telehealth for minority or other populations was not uniformly reimbursed, frequently used or easily accessed by individuals [123]. Two main factors have been critical in making remote monitoring a reality in recent years: (1) the commercial availability of CGM and AID systems, with integrated software permitting the sharing of data with healthcare professionals via smartphone apps and secure web-based platforms; and (2) the COVID-19 pandemic, which forced rapid changes in practice [124, 125].

When traditional, clinic-based models of care were suddenly no longer feasible during the COVID-19 pandemic, healthcare professionals rapidly transitioned to phoning (or video calling) patients on their clinic lists and were able to access clinical data through cloud-based portals. For those who had access to CGM systems, this was of considerable value, as adjustments in insulin and other glycaemic therapies could be made based on TIR, individual day glucose profiles and estimates of HbA_1c_ based on remotely accessed data. There was also an increase in use of CGM data in inpatient settings to reduce face-to-face contact between staff and patients [126, 127].

Many of these examples of innovation have persisted since the pandemic ended and are particularly valuable for certain individuals, with available evidence showing the benefits of telemedicine [128, 129] as well as some of the gaps that remain [130]. For example, many clinics offer patients a mixed model in which some aspects of care can be addressed in remote consultations, while others require face-to-face appointments (or can vary according to ongoing needs). This can help to improve access to diabetes technology and enable tailoring of services to people with diabetes, for example those who do not wish to (or cannot) take time off work for diabetes review. Additionally, if patients are ill (e.g. have respiratory symptoms), healthcare professionals can review them remotely without personal exposure to risk.

On the other hand, some people with diabetes do not see remote review as adequate and may be keen to return to a full clinic-based model. Anecdotally, some individuals have reported being unhappy not to have ‘seen a doctor’ since before the pandemic, despite having regular remote appointments. A key disadvantage of over-reliance on remote monitoring is that certain key health parameters are not as easily measured at home, including renal function, blood pressure, cholesterol and albuminuria, as well as skin assessments and checks for eye and foot complications. Some solutions have been proposed for these issues but have not yet been widely implemented [131].

Remote monitoring can be performed in a synchronous or an asynchronous manner. Much of what is discussed above relates to the synchronous (or real-time) use of remote monitoring. However, asynchronous monitoring can allow for clinic-based protocols that use cloud-based programs to continuously monitor patients, generating alerts to clinic staff concerning glucose levels or use of devices. Wearables are becoming increasingly common in the general population for a variety of uses [132] and there are reports of successful use of this technology in cardiology [133], for monitoring high-risk diabetic feet [134] and in paediatric diabetes programmes [135], with potential for expansion into other areas.

People living in rural areas are the ideal target for telemedicine, but they may experience specific barriers, including limited internet access, fewer technological services and socioeconomic barriers. Although the evidence is still incomplete, it appears that use of telemedicine in rural areas may improve glycaemic management, increase medication adherence and improve compliance with quality indicators. 

Better results may be obtained in the future if telemedicine can be adopted into structured programmes addressing patient motivation in collaboration with local healthcare personnel [136].

Under-resourced individuals also experience barriers to accessing telemedicine, even though it may be of benefit. They may not have compatible smartphones or computers at home for data sharing and video-enabled visits. Internet access and mobile phone service may be limited, and some workplace environments (e.g. fieldwork, construction work, housekeeping, factory work) may not allow for breaks for telemedicine visits [137]. These factors contribute to the digital divide as well as to digital literacy. If people have no access to the tools, they will not learn how to use them for their healthcare.

### Individualisation

In the USA, the Food and Drug Administration (FDA) has authorised the use of CGM systems, insulin pumps and AID controllers to create an ecosystem of AID components that can be mixed and matched to fit the individual needs of people with diabetes. Nevertheless, challenges remain in the interoperability of various components, and academic and corporate groups should continue to work on developing a global interoperability standard [138].

There are several potential future directions for the development of more individualised AID systems, including better insulin time–action profiles, alternative routes of insulin delivery, novel control algorithms and incorporation of adjunct agents (e.g. glucagon, amylin, GLP-1RAs, and SGLT2 inhibitors). Additional inputs, such as motion sensing, meal detection, environmental factors and disturbance anticipation, may be employed to manage and individualise post-meal hyperglycaemia and exercise-related hypoglycaemia. Sensors that provide additional signals (e.g. active insulin, lactate, ketones) may be helpful for some people with diabetes to optimise insulin dosing relative to current needs. In addition, further technological improvements in the sizes and shapes of devices, battery life, insulin infusion sets, physical specifications, stability, and safety of data communications, and additional customisation of the AID hardware and software to suit the needs of people with diabetes, are also needed. Many lessons can be learned from people with diabetes themselves, who pushed ahead the development of ‘DIY AID’ systems [139].

A promising application of cloud databases and data science tools is the use of adaptive technologies that can ‘learn’ and personalise the response of an AID system to the individual. Preliminary work showing the potential of adaptation has already been published, and a long-term vision for an AID personalised medicine strategy has been presented [140, 141]. Direct integration of key data from CGM and/or AID systems is needed. This integration is important for ease of access by clinicians and ease of communication with people with diabetes and for population health management/case management.


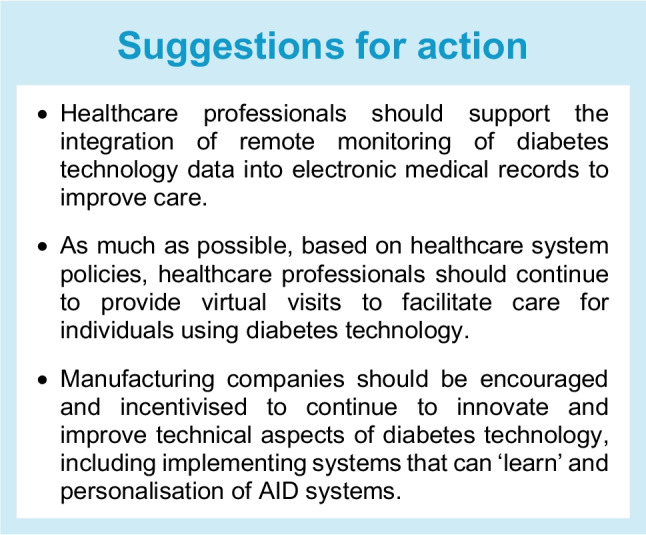


## Conclusions

It has been shown in multiple settings, including in people with type 1 and type 2 diabetes of different ages, that diabetes care and outcomes can be improved by diabetes technology. However, there are many factors to consider when implementing diabetes technology in clinical practice. Despite some diabetes technologies being available for over a decade (e.g. CGM), barriers to use still exist. In primary care settings, where many people receive their diabetes care, access to diabetes technology may be limited. Healthcare professionals using diabetes technology should remain abreast of new technology as it develops or, at a minimum, have access to colleagues to whom they can refer individuals to discuss the different options available. Taking time to educate people with diabetes and discuss available diabetes technologies in detail is essential and may help overcome perceived barriers to adoption. Joint decision making is a foundation of care, guided by individual factors. In the age of precision medicine, individualisation of care is critical, and access to diabetes technology should be facilitated for people with diabetes, with the choice of device based on their needs, preferences and skills. In particular, the availability of AID systems should be highlighted to children and adults living with type 1 diabetes in order to improve glycaemic outcomes, and with the aim of reducing disparities. Ongoing high-quality education of people with diabetes, caregivers and healthcare professionals is necessary to keep moving towards this goal. In summary, this article synthesises data on the challenges of implementing different diabetes technologies. We emphasise the need to provide practical and sustainable operational guidance to healthcare professionals, health systems, payers and regulatory agencies on how to improve the overall implementation of diabetes technology and maximise the benefits of its use.

## Data Availability

No datasets were generated or analysed in the production of this article.

## Abbreviations

AGP: Ambulatory glucose profile
AID: Automated insulin delivery
CGM: Continuous glucose monitoring
CSII: Continuous subcutaneous insulin infusion
DIY AID: Do-it-yourself AID
DT: Diabetes technology
EHR: Electronic health record
FDA: Food and Drug Administration
GLP-1RAs: Glucagon-like peptide-1 receptor agonist
IAH: Improved awareness of hypoglycaemia
MDI: Multiple daily injections
NHS: National Health Service
OTC: Over the counter
SGLT2: Sodium–glucose cotransporter 2
SMBG: Self-monitoring of blood glucose
TBR: Time below range
TIR: Time in range

## References

[1] American Diabetes Association Professional Practice Committee (2024) 7. Diabetes technology: standards of care in diabetes—2025. Diabetes Care 48(Suppl 1):S146–S66. 10.2337/dc25-S007 10.2337/dc25-S007 PMC11635043 39651978

[2] Holt RIG, DeVries JH, Hess-Fischl A et al (2021) The management of type 1 diabetes in adults. A consensus report by the American Diabetes Association (ADA) and the European Association for the Study of Diabetes (EASD). Diabetes Care 44(11):2589–2625. 10.2337/dci21-0043 34593612 10.2337/dci21-0043

[3] Sherr JL, Heinemann L, Fleming GA et al (2023) Automated insulin delivery: benefits, challenges, and recommendations. A consensus report of the joint diabetes technology working group of the European Association for the Study of Diabetes and the American Diabetes Association. Diabetologia 66(1):3–22. 10.1007/s00125-022-05744-z 36198829 10.1007/s00125-022-05744-z PMC9534591

[4] Hill-Briggs F, Fitzpatrick SL (2023) Overview of social determinants of health in the development of diabetes. Diabetes Care 46(9):1590–1598. 10.2337/dci23-0001 37354331 10.2337/dci23-0001

[5] Sjöström A, Hajdarevic S, Hörnsten Å, Isaksson U (2024) eHealth literacy and health-related internet use among Swedish primary health care visitors: cross-sectional questionnaire study. JMIR Form Res 8:e63288. 10.2196/63288 39637377 10.2196/63288 PMC11637456

[6] Tisdale RL, Purmal C, Kalwani N et al (2024) Opportunities to address specialty care deserts and the digital divide through the veterans health administration’s telehealth hub-and-spoke cardiology clinic: retrospective cohort study. J Med Internet Res 26:e53932. 10.2196/53932 39607997 10.2196/53932 PMC11638694

[7] Jeyam A, Gibb FW, McKnight JA et al (2022) Flash monitor initiation is associated with improvements in HbA(1c) levels and DKA rates among people with type 1 diabetes in Scotland: a retrospective nationwide observational study. Diabetologia 65(1):159–172. 10.1007/s00125-021-05578-1 34618177 10.1007/s00125-021-05578-1 PMC8660764

[8] Di Molfetta S, Di Gioia L, Caruso I et al (2024) Efficacy and safety of different hybrid closed loop systems for automated insulin delivery in people with type 1 diabetes: a systematic review and network meta-analysis. Diabetes Metab Res Rev 40(6):e3842. 10.1002/dmrr.3842 39298688 10.1002/dmrr.3842

[9] Fisker S, Christensen M, Bach E, Bibby BM, Hansen KW (2025) Long-term performance of two systems for automated insulin delivery in adults with type 1 diabetes: an observational study. Endocrinol Diabetes Metab 8(3):e70043. 10.1002/edm2.70043 40198839 10.1002/edm2.70043 PMC11977919

[10] Folk S, Zappe J, Wyne K, Dungan KM (2025) Comparative effectiveness of hybrid closed-loop automated insulin delivery systems among patients with type 1 diabetes. J Diabetes Sci Technol 19(5):1374–1384. 10.1177/19322968241234948 10.1177/19322968241234948 PMC11571516 38557128

[11] DigiBete (2025) Type 1 technology & resources. Available from: https://www.digibete.org/type-1-technology-resources/. Accessed: 16 Jan 2025

[12] Di Molfetta S, Rossi A, Boscari F, Irace C, Laviola L, Bruttomesso D (2024) Criteria for personalised choice of a continuous glucose monitoring system: an expert opinion. Diabetes Ther 15(11):2263–2278. 10.1007/s13300-024-01654-y 39347900 10.1007/s13300-024-01654-y PMC11467157

[13] Heinemann L, Fleming GA, Petrie JR, Holl RW, Bergenstal RM, Peters AL (2015) Insulin pump risks and benefits: a clinical appraisal of pump safety standards, adverse event reporting and research needs. A Joint Statement of the European Association for the Study of Diabetes and the American Diabetes Association Diabetes Technology Working Group. Diabetologia 58(5):862–870. 10.1007/s00125-015-3513-z 25784563 10.1007/s00125-015-3513-z

[14] Petrie JR, Peters AL, Bergenstal RM, Holl RW, Fleming GA, Heinemann L (2017) Improving the clinical value and utility of CGM systems: issues and recommendations: a joint statement of the European association for the study of diabetes and the American diabetes association diabetes technology working group. Diabetes Care 40(12):1614–1621. 10.2337/dci17-0043 29070577 10.2337/dci17-0043

[15] Sherr JL, Heinemann L, Fleming GA et al (2022) Automated insulin delivery: benefits, challenges, and recommendations. A consensus report of the joint diabetes technology working group of the European association for the study of diabetes and the American Diabetes Association. Diabetes Care 45(12):3058–3074. 10.2337/dci22-0018 10.2337/dci22-0018 36202061

[16] Conway RB, Snell-Bergeon J, Honda-Kohmo K et al (2024) Disparities in diabetes technology uptake in youth and young adults with type 1 diabetes: a global perspective. J Endocr Soc 9(1):bvae210. 10.1210/jendso/bvae210 39703363 10.1210/jendso/bvae210 PMC11655873

[17] Scottish Government (2023) Increasing access to diabetes technology. Available from: https://www.gov.scot/news/increasing-access-to-diabetes-technology. Accessed: 2 Dec 2025

[18] Nimri R, Phillip M (2025) Automated insulin delivery systems for treatment of type 1 diabetes: strategies for optimal performance. Horm Res Paediatr 98(4):371–383. 10.1159/000543654 10.1159/000543654 39864413

[19] Fan W, Deng C, Xu R et al (2025) Fully automated insulin delivery systems in type 1 diabetes: a systematic review and meta-analysis. Diabetes Obes Metab. 10.1111/dom.16499 40432359 10.1111/dom.16499

[20] Godoi A, Reis Marques I, Padrão EMH et al (2023) Glucose control and psychosocial outcomes with use of automated insulin delivery for 12 to 96 weeks in type 1 diabetes: a meta-analysis of randomised controlled trials. Diabetol Metab Syndr 15(1):190. 10.1186/s13098-023-01144-4 37759290 10.1186/s13098-023-01144-4 PMC10537468

[21] Bismuth E, Joubert M, Renard E et al (2025) Practical implementation of automated insulin delivery systems in 2025: a French position statement update. Diabetes Metab 51(3):101637. 10.1016/j.diabet.2025.101637 40073966 10.1016/j.diabet.2025.101637

[22] Beck RW, Riddlesworth TD, Ruedy K et al (2017) Continuous glucose monitoring versus usual care in patients with type 2 diabetes receiving multiple daily insulin injections: a randomized trial. Ann Intern Med 167(6):365–374. 10.7326/M16-2855 28828487 10.7326/M16-2855

[23] Martens T, Beck RW, Bailey R et al (2021) Effect of continuous glucose monitoring on glycemic control in patients with type 2 diabetes treated with basal insulin: a randomized clinical trial. JAMA 325(22):2262–2272. 10.1001/jama.2021.7444 34077499 10.1001/jama.2021.7444 PMC8173473

[24] Layne JE, Jepson LH, Carite AM, Parkin CG, Bergenstal RM (2024) Long-term improvements in glycemic control with dexcom CGM use in adults with noninsulin-treated type 2 diabetes. Diabetes Technol Ther 26(12):925–931. 10.1089/dia.2024.0197 10.1089/dia.2024.0197 38904213

[25] Ajjan RA, Battelino T, Cos X et al (2024) Continuous glucose monitoring for the routine care of type 2 diabetes mellitus. Nat Rev Endocrinol 20(7):426–440. 10.1038/s41574-024-00973-1 38589493 10.1038/s41574-024-00973-1

[26] Ajjan RA, Seidu S, Riveline JP (2024) Perspective of continuous glucose monitoring-based interventions at the various stages of type 2 diabetes. Diabetes Ther 15(8):1657–1672. 10.1007/s13300-024-01607-5 10.1007/s13300-024-01607-5 PMC11263446 38907936

[27] Seidu S, Kunutsor SK, Ajjan RA, Choudhary P (2024) Efficacy and safety of continuous glucose monitoring and intermittently scanned continuous glucose monitoring in patients with type 2 diabetes: a systematic review and meta-analysis of interventional evidence. Diabetes Care 47(1):169–179. 10.2337/dc23-1520 38117991 10.2337/dc23-1520

[28] Spartano NL, Sultana N, Lin H et al (2025) Defining continuous glucose monitor time in range in a large, community-based cohort without diabetes. J Clin Endocrinol Metab 110(4):1128–1134. 10.1210/clinem/dgae626 10.1210/clinem/dgae626 PMC11913108 39257191

[29] Zahalka SJ, Galindo RJ, Shah VN, Low Wang CC (2024) Continuous glucose monitoring for prediabetes: what are the best metrics? J Diabetes Sci Technol 18(4):835–846. 10.1177/19322968241242487 38629784 10.1177/19322968241242487 PMC11307227

[30] Basiri R, Cheskin LJ (2024) Personalized nutrition therapy without weight loss counseling produces weight loss in individuals with prediabetes who are overweight/obese: a randomized controlled trial. Nutrients 16(14):2218. 10.3390/nu16142218 39064661 10.3390/nu16142218 PMC11280332

[31] Richardson KM, Schembre SM, da Silva V et al (2024) Adding a brief continuous glucose monitoring intervention to the national diabetes prevention program: a multimethod feasibility study. J Diabetes Res 2024:7687694. 10.1155/2024/7687694 38919262 10.1155/2024/7687694 PMC11199067

[32] Dehghani Zahedani A, Shariat Torbaghan S, Rahili S et al (2021) Improvement in glucose regulation using a digital tracker and continuous glucose monitoring in healthy adults and those with type 2 diabetes. Diabetes Ther 12(7):1871–1886. 10.1007/s13300-021-01081-3 34047962 10.1007/s13300-021-01081-3 PMC8266934

[33] Uhl S, Choure A, Rouse B, Loblack A, Reaven P (2024) Effectiveness of continuous glucose monitoring on metrics of glycemic control in type 2 diabetes mellitus: a systematic review and meta-analysis of randomized controlled trials. J Clin Endocrinol Metab 109(4):1119–1131. 10.1210/clinem/dgad652 37987208 10.1210/clinem/dgad652

[34] Jancev M, Vissers T, Visseren FLJ et al (2024) Continuous glucose monitoring in adults with type 2 diabetes: a systematic review and meta-analysis. Diabetologia 67(5):798–810. 10.1007/s00125-024-06107-6 10.1007/s00125-024-06107-6 PMC10954850 38363342

[35] Lu J, Ying Z, Wang P, Fu M, Han C, Zhang M (2024) Effects of continuous glucose monitoring on glycaemic control in type 2 diabetes: a systematic review and network meta-analysis of randomized controlled trials. Diabetes Obes Metab 26(1):362–372. 10.1111/dom.15328 37828805 10.1111/dom.15328

[36] Ferreira ROM, Trevisan T, Pasqualotto E et al (2024) Continuous glucose monitoring systems in noninsulin-treated people with type 2 diabetes: a systematic review and meta-analysis of randomized controlled trials. Diabetes Technol Ther 26(4):252–262. 10.1089/dia.2023.0390 38090767 10.1089/dia.2023.0390

[37] Daly AB, Boughton CK, Nwokolo M et al (2023) Fully automated closed-loop insulin delivery in adults with type 2 diabetes: an open-label, single-center, randomized crossover trial. Nat Med 29(1):203–208. 10.1038/s41591-022-02144-z 10.1038/s41591-022-02144-z PMC9873557 36631592

[38] Reznik Y, Bonnemaison E, Fagherazzi G et al (2024) The use of an automated insulin delivery system is associated with a reduction in diabetes distress and improvement in quality of life in people with type 1 diabetes. Diabetes Obes Metab 26(5):1962–1966. 10.1111/dom.15462 38253867 10.1111/dom.15462

[39] Pasquel FJ, Davis GM, Huffman DM et al (2025) Automated insulin delivery in adults with type 2 diabetes: a nonrandomized clinical trial. JAMA Netw Open 8(2):e2459348. 10.1001/jamanetworkopen.2024.59348 39951268 10.1001/jamanetworkopen.2024.59348 PMC11829226

[40] Davis GM, Peters AL, Bode BW et al (2025) Glycaemic outcomes in adults with type 2 diabetes over 34 weeks with the Omnipod® 5 automated insulin delivery system. Diabetes Obes Metab 27(1):143–154. 10.1111/dom.15993 39382001 10.1111/dom.15993 PMC11618232

[41] Levy CJ, Raghinaru D, Kudva YC et al (2024) Beneficial effects of control-IQ automated insulin delivery in basal-bolus and basal-only insulin users with type 2 diabetes. Clin Diabetes 42(1):116–124. 10.2337/cd23-0025 38230336 10.2337/cd23-0025 PMC10788662

[42] Kudva YC, Raghinaru D, Lum JW et al (2025) A randomized trial of automated insulin delivery in type 2 diabetes. N Engl J Med. 10.1056/NEJMoa2415948 40105270 10.1056/NEJMoa2415948

[43] Bhargava A, Bergenstal RM, Warren ML et al (2025) Safety and effectiveness of MiniMed(TM) 780G advanced hybrid closed-loop insulin intensification in adults with insulin-requiring type 2 diabetes. Diabetes Technol Ther 27(5):366–375. 10.1089/dia.2024.0586 39912797 10.1089/dia.2024.0586

[44] Sherr JL, Laffel LM, Liu J et al (2024) Severe hypoglycemia and impaired awareness of hypoglycemia persist in people with type 1 diabetes despite use of diabetes technology: results from a cross-sectional survey. Diabetes Care 47(6):941–947. 10.2337/dc23-1765 38295397 10.2337/dc23-1765 PMC11116910

[45] Berry SA, Goodman I, Heller S, Iqbal A (2025) The impact of technology on impaired awareness of hypoglycaemia in type 1 diabetes. Ther Adv Endocrinol Metab 16:20420188251346260. 10.1177/20420188251346260 40520684 10.1177/20420188251346260 PMC12165273

[46] Maltese G, McAuley SA, Trawley S, Sinclair AJ (2024) Ageing well with diabetes: the role of technology. Diabetologia 67(10):2085–102. 10.1007/s00125-024-06240-2 39138689 10.1007/s00125-024-06240-2 PMC11446974

[47] Huang ES, Sinclair A, Conlin PR et al (2023) The growing role of technology in the care of older adults with diabetes. Diabetes Care 46(8):1455–1463. 10.2337/dci23-0021 37471606 10.2337/dci23-0021 PMC10369127

[48] Pratley RE, Kanapka LG, Rickels MR et al (2020) Effect of continuous glucose monitoring on hypoglycemia in older adults with type 1 diabetes: a randomized clinical trial. JAMA 323(23):2397–2406. 10.1001/jama.2020.6928 32543682 10.1001/jama.2020.6928 PMC7298607

[49] Adolfsson P, Hartvig NV, Kaas A, Moller JB, Hellman J (2020) Increased time in range and fewer missed bolus injections after introduction of a smart connected insulin pen. Diabetes Technol Ther 22(10):709–718. 10.1089/dia.2019.0411 10.1089/dia.2019.0411 PMC7591375 32003590

[50] McAuley SA, Trawley S, Vogrin S et al (2022) Closed-loop insulin delivery versus sensor-augmented pump therapy in older adults with type 1 diabetes (ORACL): a randomized, crossover trial. Diabetes Care 45(2):381–390. 10.2337/dc21-1667 34844995 10.2337/dc21-1667

[51] Boughton CK, Hartnell S, Thabit H et al (2022) Hybrid closed-loop glucose control compared with sensor augmented pump therapy in older adults with type 1 diabetes: an open-label multicentre, multinational, randomised, crossover study. Lancet Healthy Longev 3(3):e135–e142. 10.1016/s2666-7568(22)00005-8 35359882 10.1016/S2666-7568(22)00005-8 PMC8967297

[52] Kudva YC, Henderson RJ, Kanapka LG et al (2025) Automated insulin delivery in older adults with type 1 diabetes. NEJM Evid 4(1):EVIDoa2400200. 10.1056/EVIDoa2400200 39714936 10.1056/EVIDoa2400200 PMC11840810

[53] Idrees T, Castro-Revoredo I, Kantipudi S, Umpierrez G (2025) Managing diabetes in older adults: current approaches in long-term care facilities. Curr Diab Rep 25(1):27. 10.1007/s11892-025-01583-5 40138097 10.1007/s11892-025-01583-5 PMC12221204

[54] Bashir A, Joseph N, Hammond JS et al (2025) Evaluating continuous glucose monitoring after total pancreatectomy with or without islet autotransplantation: a scoping systematic review. Pancreas 54(3):e268–e277. 10.1097/mpa.0000000000002424 39626176 10.1097/MPA.0000000000002424

[55] Rizzi A, Tartaglione L, Di Leo M, Alfieri S, Pitocco D (2021) Advanced hybrid closed-loop system: first successful clinical case after total pancreatectomy. Acta Diabetol 58(7):967–969. 10.1007/s00592-021-01715-9 33864123 10.1007/s00592-021-01715-9 PMC8187211

[56] van Veldhuisen CL, Latenstein AEJ, Blauw H et al (2022) Bihormonal artificial pancreas with closed-loop glucose control vs current diabetes care after total pancreatectomy: a randomized clinical trial. JAMA Surg 157(10):950–957. 10.1001/jamasurg.2022.3702 36069928 10.1001/jamasurg.2022.3702 PMC9453632

[57] Scully KJ, Palani G, Zheng H, Moheet A, Putman MS (2022) The effect of control IQ hybrid closed loop technology on glycemic control in adolescents and adults with cystic fibrosis-related diabetes. Diabetes Technol Ther 24(6):446–452. 10.1089/dia.2021.0354 35020476 10.1089/dia.2021.0354 PMC9208855

[58] Kumar S, Soldatos G, Ranasinha S, Teede H, Pallin M (2023) Continuous glucose monitoring versus self-monitoring of blood glucose in the management of cystic fibrosis related diabetes: a systematic review and meta-analysis. J Cyst Fibros 22(1):39–49. 10.1016/j.jcf.2022.07.013 35906171 10.1016/j.jcf.2022.07.013

[59] Sherwood JS, Castellanos LE, O’Connor MY et al (2024) Randomized trial of the insulin-only iLet bionic pancreas for the treatment of cystic fibrosis- related diabetes. Diabetes Care 47(1):101–108. 10.2337/dc23-1411 37874987 10.2337/dc23-1411 PMC10733649

[60] American Diabetes Association Professional Practice Committee (2024) 9. Pharmacologic approaches to glycemic treatment: standards of care in diabetes—2025. Diabetes Care 48(Suppl 1):S181–S206. 10.2337/dc25-S009 10.2337/dc25-S009 PMC11635045 39651989

[61] Perrier Q, Lablanche S, Rakotoarisoa L et al (2025) Automated insulin delivery after beta-cell replacement failure in people living with type 1 diabetes. Diabetes Metab 51(4):101654. 10.1016/j.diabet.2025.101654 40268161 10.1016/j.diabet.2025.101654

[62] Asgharzadeh A, Patel M, Connock M et al (2024) Hybrid closed-loop systems for managing blood glucose levels in type 1 diabetes: a systematic review and economic modelling. Health Technol Assess 28(80):1–190. 10.3310/jypl3536 39673446 10.3310/JYPL3536 PMC11664472

[63] Adolfsson P, Heringhaus A, Sjunnesson K, Mehkri L, Bolin K (2024) Cost-effectiveness of the tandem t: Slim X2 with control-IQ technology automated insulin delivery system in children and adolescents with type 1 diabetes in Sweden. Diabet Med 41(11):e15432. 10.1111/dme.15432 39239975 10.1111/dme.15432

[64] Hirsch IB, Burugapalli BS, Brandner L et al (2024) Impact of continuous glucose monitoring on emergency department visits and all-cause hospitalization rates among Medicaid beneficiaries with type 2 diabetes treated with multiple daily insulin or basal insulin therapy. J Manag Care Spec Pharm 30(10-b Suppl):S21-S29. 10.18553/jmcp.2024.30.10-b.s21 10.18553/jmcp.2024.30.10-b.s21 PMC11443977 39347973

[65] Hannah KL, Nemlekar PM, Green CR, Norman GJ (2024) Reduction in diabetes-related hospitalizations and medical costs after Dexcom G6 continuous glucose monitor initiation in people with type 2 diabetes using intensive insulin therapy. Adv Ther 41(6):2299–2306. 10.1007/s12325-024-02851-8 38619722 10.1007/s12325-024-02851-8 PMC11133133

[66] Harris S, Cimino S, Nguyen Y, Szafranski K, Poon Y (2024) Cost-effectiveness of freestyle libre for glucose self-management among people with diabetes mellitus: a Canadian private payer perspective. Diabetes Ther 16(2):169–186. 10.1007/s13300-024-01677-5 10.1007/s13300-024-01677-5 PMC11794756 39688778

[67] Guerci B, Roussel R, Levrat-Guillen F et al (2023) Important decrease in hospitalizations for acute diabetes events following freestyle libre system initiation in people with type 2 diabetes on basal insulin therapy in France. Diabetes Technol Ther 25(1):20–30. 10.1089/dia.2022.0271 36094418 10.1089/dia.2022.0271

[68] Nathanson D, Eeg-Olofsson K, Spelman T et al (2025) Intermittently scanned continuous glucose monitoring compared with blood glucose monitoring is associated with lower HbA(1c) and a reduced risk of hospitalisation for diabetes-related complications in adults with type 2 diabetes on insulin therapies. Diabetologia 68(1):41–51. 10.1007/s00125-024-06289-z 39460755 10.1007/s00125-024-06289-z PMC11663194

[69] Eeg-Olofsson K, Nathanson D, Spelman T et al (2024) Initiation of intermittently scanned continuous glucose monitoring is associated with reduced hospitalization for acute diabetes events and cardiovascular complications in adults with type 1 diabetes. Diabetes Care 47(12):2164–2171. 10.2337/dc24-0690 39316385 10.2337/dc24-0690

[70] Johnson SR, Holmes-Walker DJ, Chee M et al (2022) Universal subsidized continuous glucose monitoring funding for young people with type 1 diabetes: uptake and outcomes over 2 years, a population-based study. Diabetes Care 45(2):391–397. 10.2337/dc21-1666 34872983 10.2337/dc21-1666 PMC8914416

[71] Pease AJ, Zoungas S, Callander E et al (2022) Nationally subsidized continuous glucose monitoring: a cost-effectiveness analysis. Diabetes Care 45(11):2611–2619. 10.2337/dc22-0951 36162008 10.2337/dc22-0951

[72] Biester T, Berget C, Boughton C et al (2024) International Society for Pediatric and Adolescent Diabetes clinical practice consensus guidelines 2024: diabetes technologies: insulin delivery. Hormone Res Paediatr 2024:97(6):636–662. 10.1159/000543034 10.1159/000543034 PMC11854989 39657603

[73] Simonson GD, Holt EH, Grady M, Hurrell G, Gaudiani LM, Bergenstal RM (2024) Unleashing the potential of blood glucose monitoring data with the ambulatory glucose profile report. Clin Diabetes 42(4):550–560. 10.2337/cd23-0092 39429460 10.2337/cd23-0092 PMC11486858

[74] Martens TW, Simonson GD, Bergenstal RM (2024) Using continuous glucose monitoring data in daily clinical practice. Cleve Clin J Med 91(10):611–620. 10.3949/ccjm.91a.23090 39353661 10.3949/ccjm.91a.23090

[75] Loomba L, Bonanno S, Arellano D, Crossen S, Glaser N (2023) Disparities in insulin pump use among Spanish-speaking children with type 1 diabetes compared to their non-Hispanic white peers: mixed methods study. JMIR Diabetes 8:e45890. 10.2196/45890 37294607 10.2196/45890 PMC10334715

[76] Walker AF, Haller MJ, Addala A et al (2024) Not all healthcare inequities in diabetes are equal: a comparison of two medically underserved cohorts. BMJ Open Diabetes Res Care 12(4):e004229. 10.1136/bmjdrc-2024-004229 39242122 10.1136/bmjdrc-2024-004229 PMC11381725

[77] Addala A, Hanes S, Naranjo D, Maahs DM, Hood KK (2021) Provider implicit bias impacts pediatric type 1 diabetes technology recommendations in the united states: findings from the gatekeeper study. J Diabetes Sci Technol:19322968211006476. 10.1177/19322968211006476 10.1177/19322968211006476 PMC8442183 33858206

[78] Addala A, Auzanneau M, Miller K et al (2021) A decade of disparities in diabetes technology use and hba1c in pediatric type 1 diabetes: a transatlantic comparison. Diabetes Care 44(1):133–140. 10.2337/dc20-0257 32938745 10.2337/dc20-0257 PMC8162452

[79] Odugbesan O, Addala A, Nelson G et al (2022) Implicit racial-ethnic and insurance-mediated bias to recommending diabetes technology: insights from T1D exchange multicenter pediatric and adult diabetes provider cohort. Diabetes Technol Ther 24(9):619–627. 10.1089/dia.2022.0042 35604789 10.1089/dia.2022.0042 PMC9422789

[80] Lipman TH, Smith JA, Patil O, Willi SM, Hawkes CP (2021) Racial disparities in treatment and outcomes of children with type 1 diabetes. Pediatr Diabetes 22(2):241–248. 10.1111/pedi.13139 33871154 10.1111/pedi.13139

[81] Patel PM, Thomas D, Liu Z, Aldrich-Renner S, Clemons M, Patel BV (2024) Systematic review of disparities in continuous glucose monitoring and insulin pump utilization in the United States: Key themes and evidentiary gaps. Diabetes Obes Metab 26(10):4293–4301. 10.1111/dom.15774 39010293 10.1111/dom.15774

[82] Wallia A, Agarwal S, Owen AL et al (2024) Disparities in continuous glucose monitoring among patients receiving care in federally qualified health centers. JAMA Netw Open 7(11):e2445316. 10.1001/jamanetworkopen.2024.45316 39576644 10.1001/jamanetworkopen.2024.45316 PMC11584923

[83] Prahalad P, Ebekozien O, Alonso GT et al (2021) Multi-clinic quality improvement initiative increases continuous glucose monitoring use among adolescents and young adults with type 1 diabetes. Clin Diabetes 39(3):264–271. 10.2337/cd21-0026 34421201 10.2337/cd21-0026 PMC8329017

[84] Mathias P, Mahali LP, Agarwal S (2023) Erratum. Targeting technology in underserved adults with type 1 diabetes: effect of diabetes practice transformations on improving equity in CGM prescribing behaviors. Diabetes Care 2022;45:2231-2237. Diabetes Care 46(1):222. 10.2337/dc23-er01 10.2337/dc22-0555 PMC9649356 36054022

[85] Beldon C, Rogers K, Johnson A et al (2024) Assessment of a community pharmacist remote monitoring service in patients using continuous glucose monitors. J Am Pharm Assoc (2003) 64(4s):102106. 10.1016/j.japh.2024.102106 38663533 10.1016/j.japh.2024.102106

[86] Weinstock RS, Raghinaru D, Gal RL et al (2024) Older adults benefit from virtual support for continuous glucose monitor use but require longer visits. J Diabetes Sci Technol:19322968241294250. 10.1177/19322968241294250 10.1177/19322968241294250 PMC11571625 39487727

[87] Jafar Z, Quick JD, Rimányi E, Musuka G (2024) Social media and digital inequity: reducing health inequities by closing the digital divide. Int J Environ Res Public Health 21(11):1420. 10.3390/ijerph21111420 39595687 10.3390/ijerph21111420 PMC11593574

[88] Kim DS, Eltahir AA, Ngo S, Rodriguez F (2024) Bridging the gap: how accounting for social determinants of health can improve digital health equity in cardiovascular medicine. Curr Atheroscler Rep 27(1):9. 10.1007/s11883-024-01249-9 39576395 10.1007/s11883-024-01249-9 PMC12499349

[89] Reid LJ, Gibb FW, Colhoun H et al (2021) Continuous subcutaneous insulin infusion therapy is associated with reduced retinopathy progression compared with multiple daily injections of insulin. Diabetologia 64(8):1725–1736. 10.1007/s00125-021-05456-w 33966091 10.1007/s00125-021-05456-w PMC8245368

[90] Akturk HK, Snell-Bergeon J, Shah VN (2023) Health care professionals’ perspectives on use of diabetes technologies for managing visually impaired patients with diabetes. J Diabetes Sci Technol 17(6):1610–1613. 10.1177/19322968221101629 35658590 10.1177/19322968221101629 PMC10658667

[91] Ekhlaspour L, Town M, Raghinaru D, Lum JW, Brown SA, Buckingham BA (2022) Glycemic outcomes in baseline hemoglobin A1C subgroups in the international diabetes closed-loop trial. Diabetes Technol Ther 24(8):588–591. 10.1089/dia.2021.0524 35020488 10.1089/dia.2021.0524 PMC9353995

[92] Forlenza GP, Breton MD, Kovatchev BP (2021) Candidate selection for hybrid closed loop systems. Diabetes Technol Ther 23(11):760–762. 10.1089/dia.2021.0217 34129375 10.1089/dia.2021.0217

[93] Podwojniak A, Flemming J, Tan IJ, Ghani H, Neubauer Z, Jones A (2024) Cutaneous adverse effects from diabetes devices in pediatric patients with type 1 diabetes mellitus: systematic review. JMIR Dermatol 7:e59824. 10.2196/59824 39622650 10.2196/59824 PMC11587996

[94] Passanisi S, Galletta F, Bombaci B et al (2024) Device-related skin reactions increase emotional burden in youths with type 1 diabetes and their parents. J Diabetes Sci Technol 18(6):1293–1299. 10.1177/19322968241253285 38804535 10.1177/19322968241253285 PMC11535255

[95] Diedisheim M, Pecquet C, Julla JB et al (2023) Prevalence and description of the skin reactions associated with adhesives in diabetes technology devices in an adult population: results of the CUTADIAB study. Diabetes Technol Ther 25(4):279–286. 10.1089/dia.2022.0513 36763338 10.1089/dia.2022.0513

[96] Rohner DG, Burget L, Henzen C, Fischli S (2024) Impact on diabetes control and patient-reported outcomes of a newer implantable continuous glucose monitoring system (Eversense® CGM System): a single-centre retro- and prospective observational study. Swiss Med Wkly 154:3366. 10.57187/s.3366 38579290 10.57187/s.3366

[97] Tian T, Aaron RE, Huang J et al (2023) Lipohypertrophy and insulin: an update from the diabetes technology society. J Diabetes Sci Technol 17(6):1711–1721. 10.1177/19322968231187661 37555266 10.1177/19322968231187661 PMC10658672

[98] Hirsch IB, Khakpour D, Joseph J et al (2024) The DERMIS study: methodologies, results, and implications for the future. J Diabetes Sci Technol:19322968241298005. 10.1177/19322968241298005 10.1177/19322968241298005 PMC11618839 39633523

[99] Messer LH, Berget C, Beatson C, Polsky S, Forlenza GP (2018) Preserving skin integrity with chronic device use in diabetes. Diabetes Technol Ther 20(S2):S254-s264. 10.1089/dia.2018.0080 29916740 10.1089/dia.2018.0080 PMC6011799

[100] Panther (n.d.) Diabetes technology deciphered. Available from: https://www.pantherprogram.org/. Accessed: 11 Jan 2025

[101] Kubiak T, Priesterroth L, Barnard-Kelly KD (2020) Psychosocial aspects of diabetes technology. Diabet Med 37(3):448–454. 10.1111/dme.14234 31943354 10.1111/dme.14234

[102] Zare Dehnavi A, Elmitwalli I, Alsharif HOH et al (2024) Effects of ADHD and ADHD treatment on glycemic management in type 1 diabetes: a systematic review and meta-analysis of observational studies. Diabetes Res Clin Pract 209:111566. 10.1016/j.diabres.2024.111566 38360095 10.1016/j.diabres.2024.111566

[103] Barnard-Kelly KD, Martínez-Brocca MA, Glatzer T, Oliver N (2024) Identifying the deficiencies of currently available CGM to improve uptake and benefit. Diabet Med 41(8):e15338. 10.1111/dme.15338 38736324 10.1111/dme.15338

[104] Pauley ME, Berget C, Messer LH, Forlenza GP (2021) Barriers to uptake of insulin technologies and novel solutions. Med Devices (Auckl) 14:339–354. 10.2147/MDER.S312858 34803408 10.2147/MDER.S312858 PMC8594891

[105] Commissariat PV, Boyle CT, Miller KM et al (2017) Insulin pump use in young children with type 1 diabetes: sociodemographic factors and parent-reported barriers. Diabetes Technol Ther 19(6):363–369. 10.1089/dia.2016.0375 28581817 10.1089/dia.2016.0375 PMC6435342

[106] Garza KP, Weil LEG, Anderson LM et al (2020) You, me, and diabetes: Intimacy and technology among adults with T1D and their partners. Fam Syst Health 38(4):418–27. 10.1037/fsh0000485 33591783 10.1037/fsh0000485

[107] Polonsky WH, Hood KK, Levy CJ et al (2022) How introduction of automated insulin delivery systems may influence psychosocial outcomes in adults with type 1 diabetes: Findings from the first investigation with the Omnipod® 5 System. Diabetes Res Clin Pract 190:109998. 10.1016/j.diabres.2022.109998 35853530 10.1016/j.diabres.2022.109998 PMC10901155

[108] Ng SM, Wright NP, Yardley D et al (2024) Long-term assessment of the NHS hybrid closed-loop real-world study on glycaemic outcomes, time-in-range, and quality of life in children and young people with type 1 diabetes. BMC Med 22(1):175. 10.1186/s12916-024-03396-x 38659016 10.1186/s12916-024-03396-x PMC11044460

[109] Ng SM, Katkat N, Day H, Hubbard R, Quinn M, Finnigan L (2022) Real-world prospective observational single-centre study: hybrid closed loop improves HbA1c, time-in-range and quality of life for children, young people and their carers. Diabet Med 39(7):e14863. 10.1111/dme.14863 35488481 10.1111/dme.14863

[110] Cyranka K, Matejko B, Juza A et al (2023) Improvement of selected psychological parameters and quality of life of patients with type 1 diabetes mellitus undergoing transition from multiple daily injections and self-monitoring of blood glucose directly to the MiniMed 780G advanced hybrid closed-loop system: post hoc analysis of a randomized control study. JMIR Form Res 7:e43535. 10.2196/43535 36692945 10.2196/43535 PMC9906310

[111] Reznik Y, Carvalho M, Fendri S et al (2024) Should people with type 2 diabetes treated by multiple daily insulin injections with home health care support be switched to hybrid closed-loop? The CLOSE AP+ randomized controlled trial. Diabetes Obes Metab 26(2):622–630. 10.1111/dom.15351 37921083 10.1111/dom.15351

[112] Jalilova A, Pilan B, Demir G et al (2024) The psychosocial outcomes of advanced hybrid closed-loop system in children and adolescents with type 1 diabetes. Eur J Pediatr 183(7):3095–3103. 10.1007/s00431-024-05551-1 38661816 10.1007/s00431-024-05551-1 PMC11192657

[113] Kelly CS, Nguyen H, Chapman KS, Wolf WA (2024) The emotional burden of type 1 diabetes: a cross-sectional study to understand associations between diabetes distress and glucose metrics in adulthood. Diabet Med 41(11):e15425. 10.1111/dme.15425 39149967 10.1111/dme.15425

[114] Lorenzen JT, Madsen KP, Cleal B et al (2024) Associations between use of diabetes technology and diabetes distress: a Danish cross-sectional survey of adults with type 1 diabetes. BMJ Open 14(3):e080053. 10.1136/bmjopen-2023-080053 38531585 10.1136/bmjopen-2023-080053 PMC10966817

[115] Snoek FJ, Anarte-Ortiz MT, Anderbro T et al (2024) Roles and competencies of the clinical psychologist in adult diabetes care-A consensus report. Diabet Med 41(5):e15312. 10.1111/dme.15312 38385984 10.1111/dme.15312

[116] Natale P, Chen S, Chow CK et al (2023) Patient experiences of continuous glucose monitoring and sensor-augmented insulin pump therapy for diabetes: a systematic review of qualitative studies. J Diabetes 15(12):1048–1069. 10.1111/1753-0407.13454 37551735 10.1111/1753-0407.13454 PMC10755613

[117] Fallon C, Jones E, Oliver N, Reddy M, Avari P (2022) The impact of socio-economic deprivation on access to diabetes technology in adults with type 1 diabetes. Diabet Med 39(10):e14906. 10.1111/dme.14906 35751860 10.1111/dme.14906 PMC9544624

[118] Agarwal S, Simmonds I, Myers AK (2022) The use of diabetes technology to address inequity in health outcomes: limitations and opportunities. Curr Diab Rep 22(7):275–281. 10.1007/s11892-022-01470-3 35648277 10.1007/s11892-022-01470-3 PMC9157044

[119] Pickup JC, Yemane N, Brackenridge A, Pender S (2014) Nonmetabolic complications of continuous subcutaneous insulin infusion: a patient survey. Diabetes Technol Ther 16(3):145–149. 10.1089/dia.2013.0192 24180294 10.1089/dia.2013.0192 PMC3934434

[120] Deiss D, Adolfsson P, Alkemade-van ZM et al (2016) Insulin infusion set use: european perspectives and recommendations. Diabetes Technol Ther 18(9):517–524. 10.1089/dia.2016.07281.sf 27526329 10.1089/dia.2016.07281.sf PMC5040072

[121] Phillip M, Nimri R, Bergenstal RM et al (2023) Consensus recommendations for the use of automated insulin delivery technologies in clinical practice. Endocr Rev 44(2):254–280. 10.1210/endrev/bnac022 36066457 10.1210/endrev/bnac022 PMC9985411

[122] Carić B, Marin S, Malinović-Pančić J, Malešević G, Mirnić D (2024) The success of insulin pump therapy: importance of education of patients and health professionals. Front Clin Diabetes Healthc 5:1464365. 10.3389/fcdhc.2024.1464365 39611060 10.3389/fcdhc.2024.1464365 PMC11602451

[123] Jang M, Johnson CM, D’Eramo-Melkus G, Vorderstrasse AA (2018) Participation of racial and ethnic minorities in technology-based interventions to self-manage type 2 diabetes: a scoping review. J Transcult Nurs 29(3):292–307. 10.1177/1043659617723074 28826353 10.1177/1043659617723074

[124] Kerr D, Warshaw H (2020) Clouds and silver linings: COVID-19 pandemic is an opportune moment to democratize diabetes care through telehealth. J Diabetes Sci Technol 14(6):1107–1110. 10.1177/1932296820963630 33050727 10.1177/1932296820963630 PMC7645128

[125] Sarwal A, Lim J, Sarwal A (2024) Telemedicine for the underserved racial and ethnic minorities during COVID-19 and beyond. Telemed J E Health 30(6):1588–1593. 10.1089/tmj.2023.0270 38739446 10.1089/tmj.2023.0270

[126] Galindo RJ, Aleppo G, Klonoff DC et al (2020) Implementation of continuous glucose monitoring in the hospital: emergent considerations for remote glucose monitoring during the COVID-19 pandemic. J Diabetes Sci Technol 14(4):822–832. 10.1177/1932296820932903 32536205 10.1177/1932296820932903 PMC7673156

[127] Gothong C, Singh LG, Satyarengga M, Spanakis EK (2022) Continuous glucose monitoring in the hospital: an update in the era of COVID-19. Curr Opin Endocrinol Diabetes Obes 29(1):1–9. 10.1097/med.0000000000000693 34845159 10.1097/MED.0000000000000693 PMC8711300

[128] Aleppo G, Gal RL, Raghinaru D et al (2023) Comprehensive telehealth model to support diabetes self-management. JAMA Netw Open 6(10):e2336876. 10.1001/jamanetworkopen.2023.36876 37792375 10.1001/jamanetworkopen.2023.36876 PMC10551767

[129] Hood K, Bergenstal RM, Cushman T et al (2025) Patient-reported outcomes improve with a virtual diabetes care model that includes continuous glucose monitoring. Telemed J E Health 31(1):75–84. 10.1089/tmj.2024.0093 10.1089/tmj.2024.0093 39166322

[130] Fogliazza F, Sambati V, Iovane B, Lazzeroni P, Street ME, Esposito S (2024) Telemedicine for managing type 1 diabetes in children and adolescents before and after the COVID-19 pandemic. J Clin Med 13(23):7359. 10.3390/jcm13237359 39685817 10.3390/jcm13237359 PMC11642187

[131] Dardari D, Franc S, Charpentier G et al (2023) Hospital stays and costs of telemedical monitoring versus standard follow-up for diabetic foot ulcer: an open-label randomised controlled study. Lancet Reg Health Eur 32:100686. 10.1016/j.lanepe.2023.100686 37520145 10.1016/j.lanepe.2023.100686 PMC10384180

[132] Wettstein R, Sedaghat-Hamedani F, Heinze O et al (2024) A remote patient monitoring system with feedback mechanisms using a smartwatch: concept, implementation, and evaluation based on the activeDCM randomized controlled trial. JMIR Mhealth Uhealth 12:e58441. 10.2196/58441 39365164 10.2196/58441 PMC11624455

[133] Ontario HQ (2018) Remote monitoring of implantable cardioverter-defibrillators, cardiac resynchronization therapy and permanent pacemakers: a health technology assessment. Ont Health Technol Assess Ser 18(7):1–199 PMC6235077 30443279

[134] Matijevich E, Minty E, Bray E, Bachus C, Hajizadeh M, Liden B (2024) A multi-faceted digital health solution for monitoring and managing diabetic foot ulcer risk: a case series. Sensors (Basel) 24(9):2675. 10.3390/s24092675 38732781 10.3390/s24092675 PMC11085305

[135] Prahalad P, Ding VY, Zaharieva DP et al (2022) Teamwork, targets, technology, and tight control in newly diagnosed type 1 diabetes: the pilot 4T study. J Clin Endocrinol Metab 107(4):998–1008. 10.1210/clinem/dgab859 34850024 10.1210/clinem/dgab859 PMC8947228

[136] AlQassab O, Kanthajan T, Pandey M et al (2024) Evaluating the impact of telemedicine on diabetes management in rural communities: a systematic review. Cureus 16(7):e64928. 10.7759/cureus.64928 39035595 10.7759/cureus.64928 PMC11260063

[137] Shih JJ, Kuznia M, Nouri S et al (2025) Differences in telemedicine use for patients with diabetes in an academic versus safety net health system: retrospective cohort study. J Med Internet Res 27:e64635. 10.2196/64635 40126552 10.2196/64635 PMC11976178

[138] Jendle J, Adolfsson P, Choudhary P et al (2024) A narrative commentary about interoperability in medical devices and data used in diabetes therapy from an academic EU/UK/US perspective. Diabetologia 67(2):236–245. 10.1007/s00125-023-06049-5 38041737 10.1007/s00125-023-06049-5 PMC10789828

[139] Forlenza GP, Tabatabai I, Lewis DM (2024) Point-counterpoint: the need for Do-It-Yourself (DIY) Open Source (OS) AID systems in type 1 diabetes management. Diabetes Technol Ther 26(10):689–699. 10.1089/dia.2024.0073 38669472 10.1089/dia.2024.0073

[140] Messori M, Toffanin C, Del Favero S, De Nicolao G, Cobelli C, Magni L (2019) Model individualization for artificial pancreas. Comput Methods Programs Biomed 171:133–140. 10.1016/j.cmpb.2016.06.006 10.1016/j.cmpb.2016.06.006 27424482

[141] Shi D, Dassau E, Doyle FJ 3rd (2019) Multivariate learning framework for long-term adaptation in the artificial pancreas. Bioeng Transl Med 4(1):61–74. 10.1002/btm2.10119 30680319 10.1002/btm2.10119 PMC6336673

